# The Spiritual Path of Transformation^1^

**DOI:** 10.1111/1468-5922.13096

**Published:** 2025-05-07

**Authors:** Eckhard Frick

**Affiliations:** ^1^ Munich Germany

**Keywords:** Bion, faith in “O”, Ignatius of Loyola, individuation, Jung, spirituality, transformation, transformation, individuation, spiritualité, foi en O, Jung, Bion, Ignace de Loyola, Transformation, Individuation, Spiritualität, Glaube an O, Jung, Bion, Ignatius von Loyola, trasformazione, individuazione, spiritualità, fede in O, Jung, Bion, Ignazio di Loyola, трансформация, индивидуация, духовность, вера в О, Юнг, Бион, Игнатий Лойола, transformación, individuación, espiritualidad, fe en O, Jung, Bion, Ignacio de Loyola, 转化, 自性化, 灵性, 对O的信仰, 荣格, 比昂, 依纳爵, 罗耀拉

## Abstract

Transformation is an essential component of individuation; it is not a linear but a circular process, encompassing experiences of standstill and deadlock where no longitudinal continuation is visible. Jung describes transformation in alchemical terms as a shared dialectical “reaction” between patient and analyst. The circular transformation process may require “faith in O” (Bion). Analysis may convey explicit knowledge about “O”, but the process and its goal are often implicit and unconscious. In explicitly religious or spiritual experiences, O may be called the transcendent mystery of God. In Jungian practice, the Self never manifests itself entirely. However, the analytic couple is oriented toward this uncontrollable archetype when working with dreams and when living other events in the consulting room. An archetype may seem abstract, empty, and formal‐transcendental as a Kantian condition of the possibility of symbol‐making. Conversely, we may be inflated, seized, and possessed by the archetype, which may exercise destructive and pathological qualities. The corresponding spiritualities (constructive /transformative or destructive/hindering transformation) require a “discernment of spirits”, as Jung explains with reference to Ignatius of Loyola.

## Abstract (fr)

La transformation est une composante essentielle de l'individuation; il ne s'agit pas d'un processus linéaire mais circulaire, englobant des expériences d'arrêt et d'impasse où aucune continuation longitudinale n'est visible. Jung décrit la transformation en termes alchimiques comme une « réaction » dialectique partagée entre le patient et l'analyste. Le processus de transformation circulaire peut nécessiter une « foi en O » (Bion). L'analyse peut transmettre une connaissance explicite de O, mais le processus et son objectif sont souvent implicites et inconscients. Dans les expériences explicitement religieuses ou spirituelles, O peut être conçu comme le mystère transcendant de Dieu. Dans la pratique jungienne, le Soi ne se manifeste jamais entièrement. Cependant, le couple analytique est orienté vers cet archétype indisponible lorsqu'il travaille avec les rêves et lorsqu'il vit d'autres événements dans la salle de consultation. Un archétype peut sembler abstrait, vide et formel‐transcendantal en tant que condition kantienne de la possibilité de créer des symboles. Par contre nous pouvons être inflationnés, saisis ou possédés par l'archétype, qui peut exercer des qualités destructrices et pathologiques. Les spiritualités correspondantes (transformation constructive/transformatrice ou destructive/obstacle à la transformation) requièrent un « discernement des esprits », comme l'explique Jung en se référant à Ignace de Loyola.

## Abstract (de)

Transformation ist ein wesentlicher Bestandteil der Individuation; es handelt sich nicht um einen linearen, sondern um einen zirkulären Prozess, der Erfahrungen von Stillstand und Sackgassen umfasst, bei denen keine Fortsetzung im Längsschnitt erkennbar ist. Jung beschreibt Transformation in alchemistischen Begriffen als eine gemeinsame dialektische "Reaktion" zwischen Patient und Analytiker. Der zirkuläre Transformationsprozess kann "Glauben an O" (Bion) erfordern. Die Analyse kann explizites Wissen über O vermitteln, aber der Prozess und sein Ziel sind oft implizit und unbewusst. In explizit religiösen oder spirituellen Erfahrungen kann O als das transzendente Mysterium Gottes bezeichnet werden. In der Jungianischen Praxis manifestiert sich das Selbst nie vollständig. Das analytische Paar orientiert sich jedoch an diesem unverfügbaren Archetyp, wenn es mit Träumen arbeitet und andere Ereignisse im Sprechzimmer erlebt. Ein Archetyp kann abstrakt, leer und formal‐transzendental erscheinen, als Kantsche Bedingung für die Möglichkeit der Symbolbildung. Umgekehrt können wir vom Archetyp inflationiert, ergriffen und besessen sein, was destruktive und pathologische Eigenschaften haben kann. Die entsprechenden Spiritualitäten (konstruktiv/transformativ oder destruktiv/Transformation verhindernd) erfordern eine "Unterscheidung der Geister", wie Jung mit Bezug auf Ignatius von Loyola erklärt.

## Abstract (it)

La trasformazione è una componente essenziale per l'individuazione; non è un processo lineare, bensì circolare, che comprende esperienze di blocco, di essere ad un punto morto, dove non si riesce a vedere una possibile evoluzione. Jung ha descritto questa trasformazione con una terminologia alchemica, facendo riferimento alla "reazione" dialettica tra paziente ed analista. Il processo circolare di trasformazione può richiedere di avere "fede in O" (Bion). L'analisi può offrire una conoscenza esplicita di "O", ma il processo ed i suoi obiettivi sono spesso impliciti ed inconsci. Nelle esperienze religiose o spirituali, "O" può essere il mistero della trascendenza di Dio. Nell'analisi junghiana, il Sé non si manifesta mai interamente. Eppure, la coppia analitica, quando è al lavoro con i sogni o quando vengono sperimentati eventi particolari nella stanza d'analisi, è orientata verso questo archetipo, difficile da cogliere. Un archetipo può apparire come un concetto astratto, vuoto e trascendente, come una condizione kantiana per la creazione del simbolo. Comunque, è importante osservare che possiamo essere inflazionati e dominati dall'archetipo, che può esercitare anche un potere distruttivo e patologico. Le corrispondenti esperienze spirituali (costruttivo/trasformativo o distruttivo/paralizzante) richiedono un "discernimento dello spirito", come osserva Jung in riferimento ad Ignazio di Loyola.

## Abstract (ru)

Трансформация является важным компонентом индивидуации; это не линейный, а циклический процесс, включающий переживание застоя и тупика, когда не видно дальнейшего продолжения. Юнг описывает трансформацию в алхимических терминах как общую для пациента и аналитика диалектическую «реакцию». Циркулярный процесс трансформации может потребовать «веры в О» (Бион). Анализ может предложить осознанное знание об О, но сам процесс и его цель часто являются неявными и бессознательными. В случае религиозных или духовных переживаний О может быть названо трансцендентной тайной Бога. В практике юнгианского анализа Самость никогда не проявляет себя полностью. Однако аналитическая пара ориентируется на этот непреложный архетип, когда работает со сновидениями и переживает другие события в кабинете аналитика. Архетип может казаться абстрактным, пустым и формально‐трансцендентным, как состояние возможности создания символов, по Канту. И наоборот, мы можем быть инфлированы и захвачены архетипом, и тогда могут проявляться наши деструктивные и патологические качества. Соответствующие духовные начала (конструктивное/трансформирующее или деструктивное/препятствующее трансформации) требуют «различения духов», как объясняет Юнг со ссылкой на Игнатия Лойолу.

## Abstract (es)

La transformación es un componente esencial de la individuación; no es un proceso lineal sino circular, que abarca experiencias de estancamiento y bloqueo en las que no es visible ninguna continuación longitudinal. Jung describe la transformación en términos alquímicos como una «reacción» dialéctica compartida entre el paciente y el analista. El proceso de transformación circular puede requerir «fe en O» (Bion). El análisis puede transmitir un conocimiento explícito sobre O, pero el proceso y su objetivo suelen ser implícitos e inconscientes. En las experiencias explícitamente religiosas o espirituales, O puede denominarse el misterio trascendente de Dios. En la práctica Junguiana, el Self nunca se manifiesta por completo. Sin embargo, la pareja analítica se orienta hacia este arquetipo ‐no‐disponible ‐ al trabajar con los sueños y al vivenciar otros acontecimientos en la consulta. Un arquetipo puede parecer abstracto, vacío y formal‐trascendental como condición Kantiana de la posibilidad de creación de símbolos. A la inversa, podemos sentirnos inflados, tomados y poseídos por el arquetipo, que puede ejercer cualidades destructivas y patológicas. Las espiritualidades correspondientes (constructiva/transformativa o destructiva/que obstaculiza la transformación) requieren un «discernimiento de espíritus», como explica Jung en referencia a Ignacio de Loyola.

## Abstract (zh)

转化的灵性道路

转化是自性化的重要部分；它不是线性的, 而是一个循环的过程, 包括了停滞和僵局的体验, 这些体验中看不到任何纵向的延续。荣格用炼金术的术语来描述转化, 将其视为患者和分析师之间的一种共享的辩证“反应”。循环的转化过程可能需要“对O的信仰”(比昂)。分析可能会传达关于O的明确知识, 但过程及其目标通常是隐含的和无意识的。在明确的宗教或精神体验中, O可能被称为上帝的超越之谜。在荣格的实践中, 自性从未完全显现。然而, 当分析师与患者一起处理梦境以及在咨询室中经历其他事件时, 双方都会被导向这个无法回避的原型。一个原型可能看起来是抽象的、空洞的、形式上的超越的, 是康德所说的象征产出的可能性条件。相反, 我们可能会被原型所膨胀、抓住、占据, 而原型可能会表现出破坏性和病理性的品质。相应的灵性的部分 (建设性/转化性或是破坏性的/阻碍转化的)需要“灵性的辨别”, 正如荣格通过引用依纳爵·罗耀拉所做的解释。

## Spirituality, Transformation

The author of the following lines is a Jungian analyst and a Jesuit who accompanies Ignatian retreats, “Spiritual Exercises”. First of all: What is spirituality? What is transformation? What about the material *and* spiritual stuff we fill into the Hermetic vessel, the “container” and what about the outcome of the transformative process, given that in our analytical alchemy the physical and the spiritual are linked as the alchemist links laboratory and oratory: “*in stercore invenitur*—found in filth” (Jung, 1946, para. 384)? When Jung talks in 1932 at the Alsatian Pastoral Conference, he calls it a “religious outlook”:

Among all my patients in the second half of life—that is to say, over thirty‐five—there has not been one whose problem in the last resort was not that of finding a religious outlook on life. It is safe to say that every one of them fell ill because he had lost what the living religions of every age have given to their followers, and none of them has been really healed who did not regain his religious outlook. This of course has nothing whatever to do with a particular creed or membership of a church. (Jung, 1932, para. 509)

We see that this “spiritual healing” is not religious in a confessional way; it is “spiritual”, as we use this umbrella term nowadays. But what are the milestones of this transformative process? Let’s use Bion’s formulations 2:

Within this usage I have used the term “transformation” in three ways that need to be distinguished from each other: the term “transformation” related to (i) the total operation which includes the act of transforming and the end product: for this I shall use the sign **T**; (ii) the process of transformation: sign **Tα** and (iii) the end product: sign **Tβ**.

In the session the facts of his behaviour are like the facts of a painting and from them I must find the nature of his representation (or, in terms of my notation, the nature of that which I denote by the sign **T (patient) β)**. From the analytic treatment as a whole I hope to discover from the invariants in this material what **O** is, what he does to transform **O** (that is to say, the nature of **T (patient) α)** and, consequently, the nature of **T (patient)**. This last point is the set of transformations, in the group of transformations, to which his particular transformation (**T (patient)**) is to be assigned. (Bion, 1965, pp. 14–15)

**O** is not defined, and **O** is distinct from knowledge **K**. For Bion, **O** is the Kantian thing in itself, which cannot be known but has to be supposed:

[…] spirituality fails when it is committed to pre‐defined content, when its framework no longer allows creative indetermination. An opening spirituality creates framework conditions that allow openness. Opening spirituality allows transcendent, i.e. cross‐border, queries without having to answer them exhaustively. (Küchenhoff, 2024, p. 55)

**O** is the spiritual, the mystical. “Faith in **O**” is crucial for the patient and the analyst. According to Bion (1963/2013), we may call **O** “**‐G”**, the negativity of God, provoking suffering as the absent breast provokes the infant and provokes a thought (Pickering, 2019). Bion can be understood in the context of negative or apophatic theology: We cannot (positively or in a “kataphatic”, affirmative manner) describe what God is, only what he or she or it is *not*. This suspension of theological descriptions and definitions entails a crucial therapeutic consequence: The non‐judgemental attitude towards our patients’ God‐images (Frick, 2024). Conversely, the experience of God begins when conceptual, kataphatic knowledge **K** fails. Two of Jung’s favourite mystics are representatives of negative theology (Gutschmidt & Carl, 2024): Eckhart and St John of the Cross. Consequently, **O** is not only the first milestone, the beginning of the process, but also the last milestone, the ultimate outcome. The whole process of transformation equals the transformation from **K ➔ O**. With reference to St John of the Cross’ “dark night”, the suffering from the absence of God‐experience, Bion formulates the transformation of kataphatic knowledge **K** into the apophatic experience of **O**:

Tα→Tβ=K→O.

Bion (1970/1975) repeats several times that the analyst should impose:

on himself a positive discipline of eschewing memory and desire. I do not mean that “forgetting” is enough: what is required is a positive act of refraining from memory and desire. It may be wondered what state of mind is welcome if desires and memories are not. A term that would express approximately what I need to express is “faith”—faith that there is an ultimate reality and truth—the unknown, unknowable, “formless infinite”. This must be believed of every object of which the personality can be aware: the evolution of ultimate reality (signified by **O**) has issued in objects of which the individual can be aware. (Bion, 1970/1975, p. 31)

And he ends the book:

What is to be sought is an activity that is both the restoration of god (the Mother) and the evolution of god (the formless, infinite, ineffable, non‐existent), which can be found only in the state in which there is NO memory, desire, understanding. (Bion, 1970/1975, p. 129)

Really: Just now, while writing or reading, we aim at the opposite, discussing knowledge **K** *about* **O** and mystical experience. It is true: Bion addresses this **K** and the process **O**➔**K**. But he provides the point of view (“vertex”, he says) of faith, which is so important when we consider Jung’s reflections on spiritual transformation.

**Figure 1:**
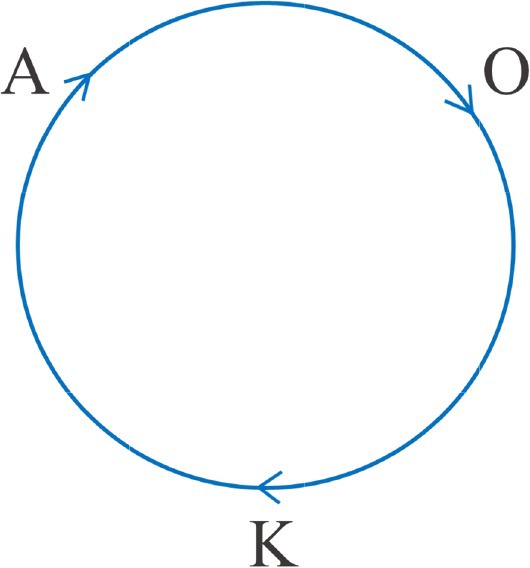
Adapted by author from Goetzmann, L. (2008). “Ueber die Verwandtschaftsbeziehungen der negativen Theologie—Transformative Transzendenz und die Erfahrung ‘O’ in der Mystik”. *Psyche—Zeitschrift für Psychoanalyse, 62*(12), 1230–1245.

Formulating equations with a sense of direction entails a linear model of progression, e.g., human development from cradle to grave, as we formulate in attachment theory. Bion’s point of view of the mystical *and* of the psychoanalytical way is circular in the following sense (Goetzmann, 2008)—see figure 1:

Apophasis is a rhetorical device wherein the speaker brings up a subject by either denying it or denying that it should be brought up: “I refuse to discuss the rumour that my opponent is a drunk”. Apophatic theology, also known as negative theology, is a form of theological thinking and religious practice which attempts to approach God, the Divine, by negation, to speak only in terms of what may not be said about the perfect goodness that is God.

Jung’s model of individuation is, too, quite different from linear process outcome designs. Vogel (2017) characterizes the process‐outcome model of individuation as a circular “therapeutical process theory”: “The therapeutic process can thus be defined as a compressed life path, as individuation under maximally favourable conditions” (Vogel, 2017, p. 119).

According to Vogel, the Jungian transformation model is not a linear change but a circular process comprising pauses, regressions, nigredo, putrification, and death, as Jung explains in “Psychology of the Transference” (*CW* 16). Despite the dimension of finality, individuation “never comes to a final resting place where one can say ‘It is done’. It is an ongoing opus, that is never final, never complete” (Stein, 2008, p. 15). Among the methods of individuation in analysis, active individuation is vital for four reasons (Vogel, 2017, pp. 120–121):

- It trains the “art of letting things happen, action through non‐action” (Jung, 1957, para. 20)
- It is the creative expression of the process of individuation
- It fosters the process of individuation by enquiring about imagined images, scenes, and figures about their meaning and relevance for the individual process
- It trains the transcendent function and, therefore, the union of opposites, divesting “the self of the false wrappings of the persona on the one hand, and of the suggestive power of primordial images on the other” (Jung, 1935, pp., para. 269).

## Imagination in Ignatian Spiritual Exercises

During the 1930s, Jung comments on Nietzsche’s Zarathustra, on Indian Yoga. He moves “on to a Western yoga form, if this word is applicable to the West at all: namely, the *Exercitia spiritualia* of Saint Ignatius of Loyola” (November 3, 1939, Liebscher, 1939/1940[2023], p. 59). During the 30s! While Jung teaches in Zürich, we know what is happening in Germany. And we remember what he says about Wotan, about *Ergriffenheit*, being seized by the archetype of the spirit. Contrary to the prevalent 'wellness' conception of spirituality, Jung’s work delves into spirituality’s deep resources and risks. This is evident in his exploration of the bipolarity of the archetype of the spirit, e.g., in his description of Nietzsche’s enthusiastic and self‐wounding inflation with the old wise man or woman, but also in Jung’s Yoga and Ignatian Exercises seminars. Jung's academic depth is further underscored by his detailed examination of the differences between the terms “spirit” and “pneuma” and their German translation “*Geist*”. According to Jung, “*Geist*” signifies effervescence, which can entail “*Begeisterung*” (enthusiasm) but which may also be destructive.

Unfortunately, Jung did not make the Exercises in their complete form of four weeks or a one‐week retreat. In his autobiography, he tells Aniela Jaffé about his uncanny childhood memories with Jesuits, an order that was forbidden by the Swiss Federal Constitution until 1973. However, he knew Hugo Rahner and other Jesuits and studied the Spiritual Exercises' text. In other words, he remains in the perspective **O➔K**, experiencing the Exercises:

[…] not, however, in the strict Catholic form, because it’s impossible for me to do that. But I did at least, to the best of my knowledge and ability, present the meditation in a manner that closely resembled the way a Jesuit would perceive it. (December 15, 1939, Liebscher, 1939/1940[2023], p. 128)

When Jung comments on imagination in the Exercises, he is not only interested in the technical, methodological aspects of what Ignatius proposes as *imaginación*. He is intrigued by the difference between an active imagination (where the unconscious manifests itself) and a more passive, guided one. In other words, does imagination open or close the door towards the uncontrollable/undisposable (“unverfügbar”, Küchenhoff, 2024; Rosa, 2020) **O**? He finds the criterion in Ignatius’ more or less orthodox or “dogmatically correct” visions and, in particular in his visions of the serpent and the cross. I quote the Reminiscences:

[19] While he was in the almshouse something happened to him, many times: in full daylight he would see clearly something in the air alongside him, which would give him much consolation, because It was very beautiful, enormously so. He couldn’t properly make out what it was an image of, but somehow it seemed to him that it had the shape of a serpent, and it had many things which shone like eyes, though they weren’t eyes. He used to take much delight in and be consoled by seeing this thing, and the more times he saw it, the more his consolation would increase. And when that thing would disappear from his sight he would feel sad about it.

[31] After this had lasted a good while, he went off to kneel at a cross which was nearby in order to give thanks to God. And there appeared to him there that vision which had often been appearing and which he had never recognized: i.e., that thing mentioned above which seemed very beautiful to him, with many eyes. But being in front of that cross he could well see that that thing of such beauty didn’t have its normal colour, and he recognized very clearly, with strong backing from his will, that it was the devil. And in this form later the devil had a habit of appearing to him, often and for a long time, and he, by way of contempt would cast it aside with a staff he used to carry in his hand.

In these passages, we encounter the beginning of the probably most crucial method of Ignatian spirituality: discernment of spirits. Already during his recovery following his wounding in the battle of Pamplona, Ignatius had observed the coming and going of *consolation* and *desolation*. Here, in Manresa, during the first retreat he makes, he perceives consolation in the presence of the snake vision and, later on, in front of the cross, desolation provoked by the same vision. In the Spiritual Exercises, he will develop a sophisticated system for discerning which soul movement is prompted by the Holy Spirit and which, on the contrary, by the Devil’s Spirit. Importantly, this discernment is not cross‐sectional, not even longitudinal; it is circular, encompassing experiences of standstill and deadlock where no longitudinal continuation is visible.

Discernment of spirits and imagination: How does it work, and why is Jung so interested in Ignatius’ visions? According to Jung, the “undogmatic” character of simple visions emerging from the unconscious indicates their authenticity. They are followed by an elaboration “with strong backing from his will”, as Ignatius says, and with a dogmatic subsumption.

Liebscher (lxvi · introduction to volume 7, 1939/1940[2023]) summarizes:

Jung’s choice to work through the Ignatian exercises in the lecture series of 1939/1940, despite his lack of expert knowledge in the field, was triggered not least by his intention to compare his visionary experiences in the years from 1913 onward to the visions of Saint Ignatius.

Jung says:

Then such visions can occur, which bring a kind of solution, rather like an epiphany. It is a concretized epiphany. The result is then very often that a conscious conviction takes root, such as the belief that God particularly helped the person by appearing right at that very moment. Psychologically, this simply means that at certain typical moments of human life, spontaneous manifestations of the unconscious occur which have this resolving effect. How such things are described is left to the discretion of the individuals and their convictions. (July 7, 1939, 1939/1940[2023], p. 39)

Jung does not explicitly use the specific term “discernment of spirits”. In German, it is: “Es ist der Weltanschauung des Einzelnen überlassen” (Jung, 1939/1990, p. 42). The English translation (“discretion”) of Jung’s Exercises‐Seminar is closer to discernment. As Jung says, visions and imaginations are actively processed by discernment, which may be more or less “dogmatic”. In this context, what he calls “dogmatic” seems to hinder “imagination”. Surprisingly but in line with that, during the Zarathustra‐Seminars he affirms about his Catholic patients “that they all suffered from a most remarkable extinction of fantasy—they had the greatest trouble about it. It was almost impossible to get them to realize a fantasy simply because they had gone through the Jesuit training, the exercises that systematically destroy the imagination” (June 24, 1936, Jarrett, 1988, p. 250).

As an example of neurobiological research in meditation, we may consult a recent study about changes in brain functional connectivity provoked by an Ignatian retreat (Wintering et al., 2021). Of course, this pre‐post‐design is not circular but absolutely linear. Let’s have a look at two of the pre‐post‐changes:

- Increased connectivity between the pallidum, the primary dopaminergic region, and the posterior cingulate could reflect how dopamine augments the self‐awareness process or a more positive outlook on the self.
- Increased connectivity was found between the posterior cingulate gyrus, the superior frontal gyrus, and the left pallidum. Both the superior frontal gyrus and posterior cingulate may be associated with self‐awareness since the posterior cingulate is a structure in the self‐processing component of the default mode network.

And the authors add: Since a substantial element of the Ignatian retreat is spent in self‐reflection and silence, increased connectivity between these structures could be consistent with such a subjective response.

## Archetype of the Spirit

Before presenting you with another example of spiritual transformation, I would like to take a break to re‐consider Jung’s central theme in the 30s, which is the archetype of the spirit. We cannot capture the spirit despite finding evidence of spiritual changes in fMRI studies or in psychological scales. Conversely, we may be seized (*ergriffen*) by the spirit or completely untouched. Jung considered Nietzsche to be seized, inflated by the archetype of the spirit, by the old wise Zarathustra. And Jung himself, observing the German abusive Wotan spirituality, tended to be touched by this inflated spirituality.

In lecture 5 (December 1, 1939), Jung talks about meditating on Christ’s passion in the Exercises, about the problem of a suffering god. And in this context, he revisits Nietzsche:

who suffered from this Western problem, wrestled with it, and was felled by it. I mean, the problem of the collapse of the church, of traditional Christianity, that unfortunately cannot be denied. […] He made the pronouncement, “God is dead.” God has died. And he didn’t realize that in proclaiming this he was actually within the dogma, that it was a Christian pronouncement. Because God has died. He is always dying, and the death of Christ is one of the mysteries of Christianity. […] But Nietzsche didn’t mean it like that; he meant: God has come to an end. With that, God coming to an end and having no successor, something very special happened for the world. Nietzsche didn’t know that *he* would become God’s successor. Then when mental illness got the better of him, he signed his letters with names such as “the crucified one” or “Dionysos”, or “Zagreus”—that is, the mutilated one, who was also a god, also a deceased god. (Liebscher, 1939/1940[2023], p. 106).

Nietzsche claimed in The Birth of Tragedy that “religions tend to die out” when “the mythical prerequisites of a religion are systematized under the strict, rational eyes of a righteously dogmatic faith as a finished sum of historical events” (Nietzsche, 1872/2023, p. 62). According to Bishop (1995), Jung marked this passage in his copy. He owes this insight to Nietzsche: Religions die by dogmatism and by reducing myths to metaphysical explanations. However, Nietzsche’s “God is dead” is much more exciting, “ecstatic”, we may say with Nietzsche: God’s death is linked to Jesus’ cross, and according to Jung, Nietzsche is inflated by becoming the dying God’s successor.

Jung underlines the psychological importance of the “autonomous” *Geist*; he treats spirit as a *phenomenon* rather than a *concept* (Gitz‐Johansen, 2020). Jung opposes the autonomous spirit to a materialistic “psychology without a soul”. However, he does not claim a spiritual bypassing of human nature, of our instinctual and bodily existence.

All people who claim to be spiritual try to get away from the fact of the body; they want to destroy it in order to be something imaginary, but they never will be that, because the body denies them; the body says otherwise. They think they can live without sex or feeding, without the ordinary human conditions; and it is a mistake, a lie, and the body denies their convictions. That is what Nietzsche means here. The Superman, the self, is the meaning of the earth; it consists of the fact that we are made of earth. Therefore, when you study symbols of individuation, you always find that no individuation can take place—I mean symbolically—without the animal, a very dark animal, coming up from primordial slime, enters the region of the spirit; that one black spot, which is the earth, is absolutely indispensable on the bright shield of spirituality. (Zarathustra‐Seminar, May 24, 1934, Jarrett, 1988, p. 64)

Confronted with the archetype of the spirit and transformation in spiritual and therapeutic processes, there are the following possible extremes of defence mechanisms:

- Spiritual bypassing: avoiding transformation by rationalizations, persona‐shaping, denial of the body, and ideologies of all kinds
- Spiritual neglect: not valorizing the other’s spiritual dimension and my own, reducing it to more or less childish needs
- Spiritual inflation: being seized, possessed by a more or less malignant spirit, often with the best intentions

All in all, spirituality requires discernment. Perhaps the best example of spiritual transformation is the following.

## Spiritus Contra Spiritum

Jung’s letter to Bill Wilson is well known. He suggests a discernment of spirits between the Devil and the Holy Spirit and comes to the famous helpful formula: *Spiritus contra spiritum*. Jung’s intervention in ***spired*** the 12 steps that are still used worldwide in AA (Alcoholics Anonymous) meetings. The spiritual AA self‐help movement is the best studied of all Jungian “therapies” despite criticism of a recent Cochrane review (Kelly et al., 2020). Additionally, it is a caveat against the “hungry ghosts” of Western civilization, searching for accelerated spiritual experiences with psychedelic drugs (Osterhold & Fernandes‐Osterhold, 2023).

However, as Ginny Hillebrand (2021) and McDonnell et al. (2024) have shown, the situation is more complex. Bill Wilson wrote a “secret” letter to Jung detailing his use of LSD and the success in treating alcoholics with LSD. We do not know whether Jung changed his opinion, given that he died before he could reply.

Another critical point: is spirituality “compulsory” in AA groups? What about the “non‐religious”? (Trombley, 2024). Let us remember that “spirituality”, as we understand it in health care, does not necessarily presuppose a religious affiliation. I quote three AA members who explain their approach to spirituality. I am grateful that these persons accepted their testimonies to be published:

Klaus: And when I say I’m an alcoholic, I’m only saying it for myself, of course. I say that so that I don’t forget it again. It’s essential for me. Of course, you can also forget it. And that’s why we always say it at the beginning. But nobody has to say it. That’s also important to know. Yes, spirituality is, of course, also a complicated topic in AA. It was for me at the beginning, too, because I couldn’t do anything with it. I had nothing to do with any gods or anything. I left the church. It was essential for me, for example, as I heard in the meeting that others at AA have also left the church. And it’s tough to explain what that is. Of course, I only got to know about it over time when I came to AA. I learned about it from other people. It’s not that someone imposed it on me or said you must do it the way I do. That’s not how it works here, but you find out for yourself what others understand God or their power to be.

Susanne: My name is Susanne. I’m an alcoholic, and I was brought up Catholic with a punishing God who saw everything, and I always had to reckon with the punishments. The nice thing for me here at the AAs is that I can let go of this punishing God for the time being, and by letting go, a space can open up, and there is also no fixed time frame for when this has to happen so that something new can emerge. I have studied various religions and world religions and would like to put together my own overall picture. That’s what I’ve done. I think that I personally have limits. When I reach them, but I haven’t reached the end yet, I need help, and I have understood from the AAs that as we understand Him, God, I can, so to speak, muddle Him along. That’s a bit flippant, but I can think it up and fulfil it according to my needs. I also need to ask for help daily in a little meditation like this. That’s very important to me because I know I’m definitely reaching my limits, and that’s what I find beautiful about this kind of spirituality, which leaves it up to me to what extent and for how long and how long I want to believe in something. Thank you.

Alex: So, I was thinking, I don’t have a proper definition of spirituality. What does that really mean or should mean? I have no idea. It comes up once in our programme, in the twelfth step, after we have experienced a spiritual awakening in these steps, without defining it in more detail, C. G. Jung once spoke of *spiritus contra spiritum*, I think?

I’m not a Latin scholar. It’s actually about the spirit, and then we always have the word God in our programme, which causes some people to stumble. And we have a more excellent definition in the second step: “a power greater than ourselves”. And I learned this on my first evening in the meeting with the people sitting at these tables, “a power greater than ourselves”. I thought they could do something about the topic of God. And I couldn’t at that time. And some people live that way. I went in search of a power greater than myself; I started with natural religions. I went through everything there was in terms of religion and came to the conclusion that it didn’t work for me. I then let go of it altogether; I had already been sober for many, many years. Then I thought, “Without God then”, because obviously, I don’t have this receptor in me for a God or a power greater than myself. I have had wonderful experiences with many people along the way, including religious people, especially Catholic monks. And I still didn’t feel that there was a God there to guide me or comfort me. After a period of abstinence from a God, I always had the feeling that there was a power greater than myself. I’ve had that almost since the very first days. I have this feeling, I am a trained scientist, I have always thought, well, God starts where I stop thinking. For my sake, that was a very arrogant attitude. And I realize that it is very soothing.

And then at some point I actually started to reduce these first three steps in our programme to three short sentences. “I can’t, you can, so do it”. That was the third step. That was my prayer for quite some time. I started referring to my higher power, often as HP, as “higher power” from the video game. Through that, we have a very intimate relationship, which has carried me a lot in life. But I also tend to forget that I have this and only use my head to assert things. For me, it’s always this area of conflict on the path I’m actually on. Today, I would say that I actually managed to do that in life, and that I did it repeatedly. Thank you.

## Creativity—Createdness

Jung comments on the Ignatian “Principle and Foundation (# 23) and especially on the sentence: *El hombre es criado* (The human person is created). His interpretation: We are anticipated by the unconscious:

If we understand the words “Creatus est homo” in this way, then of course our outlook changes considerably; because if we assume that everything that we are comes from our own consciousness, then we are entirely justified in feeling a diabolical hubris. Then we make claims such as “Where there’s a will there’s a way,” and the like, and think we are the gods of this world. But unfortunately there is a multitude of different gods, not just one, and each tries as hard as they can to be the only god, which leads to all kinds of conflicts, as we well know. […] If on the other hand we understand this “Creatus est homo” correctly, then we tell ourselves that we are something that has come about, a product, that we are anticipated. We were there and did not know it. It was “known,” only we do not know who knew it. This question must remain open. (lecture 8 [January 12, 1940], 1939/1940[2023], p. 142)

In other words, creativity is not the fruit of our conscious will but rather the acceptance of the unknown, uncontrollable unconscious. Without obeyance and humility, creativity will be destroyed by inflation:

But inasmuch as you say these creative forces are in Nietzsche or in me or anywhere else, you cause an inflation, because man does not possess creative powers, he is possessed by them. That is the truth. If he allows himself to be thoroughly possessed by them without questioning, without looking at them, there is no inflation, but the moment he splits off, when he thinks, “I am the fellow”, an inflation follows.

Question: Can it be avoided?

Dr. Jung: Only by obeying completely without attempting to look at yourself. You must be quite naïve. (May 23, 1934, Jarrett, 1988, p. 40)

On October 30, 1935, Jung comes back to the difference between inflation and creativity:

Here you have it! Only people with an inflation can assume that they create. You don't create, you are created; in creation you are created. Something makes you do it, something is working through you, you are most tremendously instrumental. Try to stop creation and see what happens. If creation were our own doing, we could say yes or no, but it is a well‐known fact that the creator cannot say yes or no; he has to create, and woe unto him if he does not. (October 30, 1935, Jarrett, 1988, p. 653)

Both in the Zarathustra and the Exercises‐Seminars, “being created” is Jung’s crucial invitation to accept the unconscious’ priority. This humble attitude towards the unconscious is required for artistic, intellectual, and therapeutic works.

This is Jung’s way of expressing Bion’s faith in **O**.

## Discussion

Transformation is often considered a linear process, from a starting point A to an endpoint Z, frequently described with a valuative component, e.g., when Ignatius talks about “those who go on from good to better, the good Angel touches such soul sweetly, lightly and gently, like a drop of water which enters into a sponge” (SE 335). Consequently, when spirituality is “infused into clinical practice” (Sperry, 2025), indications for spiritual interventions are readily admitted, and contraindications are easily eschewed (Sperry, 2025, pp. 176–177). However, a classical rule of thumb warns that “therapist‐initiated, denominationally specific, religiously explicit, and in‐session spiritual interventions are probably more risky than client‐initiated, ecumenical, religiously implicit, and out‐of‐session interventions” (Richards & Bergin, 1997, p. 253). If therapists want to “mind the gap of increased secularization and increased mental health needs among clients”, spiritual and religiously based perspectives and tools should be used in a creative, secularized, and culturally sensitive manner (Plante, 2024a), e.g., mindfulness as a modified Buddhist practice, yoga for Westerners or Ignatian Spirituality Informed Therapy (I‐SIT, Plante, 2024b). The French laicistic tradition is particularly sensitized against “sectarian” pitfalls (Berna et al., 2024).

“Secularized” neutrality in analysis may be called analysts’ “faithlessness”:

[…] if they have faith or are attached religiously to a *faith* they suspend it in favour of respect for all faiths when working in analysis with patients. I will recommend this attitude of respect as the favoured countertransference attitude to be held by the Jungian psychoanalyst who is working across and within the world’s many *faiths* in today’s multicultural contexts. (Stein, 2024, p. 875)

Suspending a personal faith presupposes, however, to be aware of my faith, and it entails—without memory, desire, understanding (Bion, 1970/1975, p. 129)—faith in **O**. The circular process of transformation encompasses experiences of standstill and deadlock, both in the psychotherapeutic and in the spiritual sense. The ambivalence of spirituality requires “discernment of spirits” (according to Ignatius) and a sober understanding of “spirit” / “*Geist*” (according to Jung, e.g., in his Zarathustra Seminar, May 12, 1937):

You see, this concept has been used so often and in such a way that most people think they know what they are talking about when they use the term *spirit,* but as a matter of fact they usually do not. We have a tendency to identify it with intellect, though the word *spirit* doesn’t denote intellect at all. Of course in English there is a certain difference, but in German there is no difference at all, because the word Geist which Nietzsche uses, is used absolutely indiscriminately for intellect, mind, and spirit. German is a very strange language, it is very primitive in that respect […]. (Jarrett, 1988, p. 1065)

Not unlike Ignatius, Jung insists on the bipolarity of the spirit archetype and on the danger of being seized and inflated by spiritual enthusiasm. Consequently, transformation should not be reduced to spiritual bypassing or spiritualization. We should not confront a patient with a purely archetypal and spiritual approach to analysis before regaining a connection to the affective realm of the personal unconscious and the personal ego‐tasks (Phillips, 2024, with reference to Kathrin Asper, 1986).

Jung’s alchemical model aims at the conjunction of oratory and laboratory, finding gold in filth or—in Ignatian terms—finding God in all things. Since Jung’s reflexions in the 30s of the last century we had to learn the “subtle” power of spiritual abuse (Johnson & VanVonderen, 1991), e.g., in spiritual communities (Keul, 2022), comprising spiritual neglect, manipulation, or violence.

These shadow aspects of spiritual accompaniment and leadership provoke significant individuation and transformation hindrances. Understanding and mentalizing these shadows will help wounded persons find their own spiritual trajectory (and not a way dominated by a guru or spiritual perpetrator). This liberating process from spiritual power abuse may pave the way towards creativity.

In particular, dreams and imaginations surprise us with a creativity we have not made ourselves, despite the importance of our agency. When we consider Roesler’s investigations into the structural analysis of dreams (Roesler, 2025, in print), the dreaming ego’s agency in dream series is a vital sign of progress. The agency is a sign of vitality not only in our daily conscious endeavours but also in dreams, imagination, and in all non‐directed phantasy thinking, which Jung contrasts with directed thinking. Finding in non‐directed phantasy thinking is finding without searching, finding by serendipity.

## Conclusion: Coming Full Circle

I have tried to show that spiritual transformation is not a linear but a circular process. Knowledge in Bion’s sense tries to grasp concepts and understand them through definitions. That is why many researchers and interested scholars put effort into the definitions of spirituality. Conversely, spiritual life is not about grasping concepts but being seized (*ergriffen*) by the spirit. Accordingly, we see the same movement in Bion’s *Faith in **O*** and the Jungian ego‐self axis. In other words, there are the active component of agency and the more passive and receptive dimension of being seized by the spirit. Transformations, on the one hand, comprise an active process of modelling attitudes, relationships, and the outer world: the dream ego’s progressive agency in the context of a dream series. On the other hand, transformation is gratitude and acceptance of createdness, as Ignatius of Loyola says, often quoted by Jung. Createdness transforms an acting subject into a spiritual person, opening themselves towards transcendence. Imagination, and even more dreaming, remind us that we are active, although not only knowing **K** but searching the unknown **O** in an apophatic way **A**. Apophatic in the tradition of negative theology reminds us that we can never control nor define God, that we cannot dispose of God. This spiritual experience can also inform our attitude as analysts.

## Footnotes

^2^
For marking his quasi‐logical signs, I put them in bold.

## References

[joap13096-bib-0001] Asper, K. (1986). Depression and the dark night of the soul. Guild of Pastoral Psychology. https://www.guildofpastoralpsychology.org.uk/resources/?keywords=&filter_author=1456&filter_post_tag=&filter_year=

[joap13096-bib-0002] Berna, F. , Mengin, A. C. , Huguelet, P. , Urbach, M. , Evrard, R. , & Fond, G. (2024). Is mindfulness practice “at risk” of increasing spirituality? Systematic review and critical analysis of a claimed effect. L’Encéphale. 10.1016/j.encep.2023.11.013 38311475

[joap13096-bib-0003] Bion, W. R. (1963/2013). The psycho‐analytic study of thinking. Psychoanalytic Quarterly, 82, 301–331. 10.1002/j.2167-4086.2013.00030.x 23580215

[joap13096-bib-0004] Bion, W. R. (1965). Transformations. Change from learning to growth. William Heinemann Medical Books.

[joap13096-bib-0005] Bion, W. R. (1970/1975). Attention and interpretation. A scientific approach to insight in psycho‐analysis and groups. Tavistock Publications.

[joap13096-bib-0006] Bishop, P. (1995). The Dionysian self. De Gruyter.

[joap13096-bib-0007] Frick, E. (2024). Gerufen oder nicht gerufen? Spiritualität in der Analytischen Psychologie. Kohlhammer. https://shop.kohlhammer.de/media/catalog/product/cache/514e9679f9fe3f9bc090528d50bf0157/9/7/978‐3‐17‐042128‐8_g.jpg

[joap13096-bib-0008] Gitz‐Johansen, T. (2020). Jung and the spirit: A review of Jung’s discussions of the phenomenon of spirit. Journal of Analytical Psychology, 65(4), 653–671. 10.1111/1468-5922.12611 32897565

[joap13096-bib-0009] Goetzmann, L. (2008). Ueber die Verwandtschaftsbeziehungen der negativen Theologie—Transformative Transzendenz und die Erfahrung “O” in der Mystik. Psyche—Zeitschrift für Psychoanalyse, 62(12), 1230–1245. https://www.klett‐cotta.de/produkt/psyche‐2008‐jg‐62‐ausgabe‐12‐t‐7831

[joap13096-bib-0010] Gutschmidt, R. , & Carl, M. (2024). The negative theology of absolute infinity: Cantor, mathematics, and humility. International Journal for Philosophy of Religion, 95(3), 233–256. 10.1007/s11153-023-09897-8

[joap13096-bib-0011] Hillebrand, B. (2021). Präsenz und Kontakt. Wege zum Menschen, 73(1), 19–27. 10.13109/weme.2021.73.1.19

[joap13096-bib-0012] Jarrett, J. L. (Ed.). (1988). Nietzsche’s *Zarathustra*: Notes of the seminar given in 1934–1939 by C. G. Jung (two volumes). Princeton University Press.

[joap13096-bib-0013] Johnson, D. , & VanVonderen, J. (1991). The subtle power of spiritual abuse. Bethany House.

[joap13096-bib-0014] Jung, C. G. (1932). Psychotherapists or the clergy. *CW* 11.

[joap13096-bib-0015] Jung, C. G. (1935). The relations between the ego and the unconscious. *CW* 7.

[joap13096-bib-0016] Jung, C. G. (1939/1990). Die Exercitia spiritualia des Ignatius von Loyola. Vorlesung, gehalten an der ETH Zürich vom 16. Juni 1939 bis 8. März 1940, innerhalb der Folge “Der Individuationsprozess”. Umschrift der stenographischen Notizen von dipl. Masch.‐Ing. ETH Eduard Sidler, erstellt von Frau Ida Baumgartner (Vol. Hs 1067:2). ETH Bibliothek, Hochschularchiv.

[joap13096-bib-0017] Jung, C. G. (1946). The psychology of the transference. *CW* 16.

[joap13096-bib-0018] Jung, C. G. (1957). Commentary on *The secret of the golden flower* . *CW* 13.

[joap13096-bib-0019] Kelly, J. F. , Humphreys, K. , & Ferri, M. (2020). Alcoholics Anonymous and other 12‐step programs for alcohol use disorder. Cochrane Database of Systematic Reviews(3). 10.1002/14651858.CD012880.pub2 PMC7065341 32159228

[joap13096-bib-0020] Keul, H. (2022). Vulnerability, vulnerance and resilience—Spiritual abuse and sexual violence in new spiritual communities. Religions, 13(5), 425.

[joap13096-bib-0021] Küchenhoff, J. (2024). Öffnet Spiritualität, die dem Unverfügbaren einen Rahmen verleiht, den Erfahrungshorizont oder verschließt sie ihn? In U. Anderssen‐Reuster , E. Frick , L. Lewandowski , & H. Will (Eds.), Neuer Fortschritt in der Geistigkeit? Psychoanalyse und Spiritualität (pp. 55–70). de Gruyter.

[joap13096-bib-0022] Liebscher, M. (Ed.). (1939/1940[2023]). Jung on Ignatius of Loyola’s Spiritual Exercises. Princeton University Press. 10.1515/9780691244600

[joap13096-bib-0023] McDonnell, R. , Moriarty, J. , Cabe, I. M. , & Higgins, E. (2024). AA, Bill Wilson, Carl Jung and LSD. Journal of Analytical Psychology, 69(4), 550–580. 10.1111/1468-5922.13027 39081090

[joap13096-bib-0024] Nietzsche, F. (1872/2023). The birth of tragedy: From the spirit of music. Weimar Press.

[joap13096-bib-0025] Osterhold, H. M. , & Fernandes‐Osterhold, G. (2023). Chasing the numinous: Hungry ghosts in the shadow of the psychedelic renaissance. Journal of Analytical Psychology, 68(4), 638–664. 37553849 10.1111/1468-5922.12949

[joap13096-bib-0026] Phillips, M. (2024). The personal and the transpersonal psyche: Human suffering, the archetypes and the clinical encounter. Journal of Analytical Psychology, 69(3), 367–388. 10.1111/1468-5922.13010 38726595

[joap13096-bib-0027] Pickering, J. (2019). The search for meaning in psychotherapy: Spiritual practice, the apophatic way and Bion. Routledge/Taylor & Francis Group. 10.4324/9781315639581

[joap13096-bib-0028] Plante, T. G. (2024a). Minding the gap: Spirituality in clinical practice during increased secularization and mental health needs. Spirituality in Clinical Practice, 11(1), 83–88. 10.1037/scp0000298

[joap13096-bib-0029] Plante, T. G. (2024b). What professional psychotherapy practice can learn from the Jesuits: Introducing Ignatian spirituality informed therapy (I‐SIT). Spirituality in Clinical Practice, 11(2), 186–194. 10.1037/scp0000365

[joap13096-bib-0030] Richards, P. S. , & Bergin, A. E. (1997). A spiritual strategy for counseling and psychotherapy. American Psychological Association. 10.1037/10241-000

[joap13096-bib-0031] Roesler, C. (in print, 2025). Dreams and dream interpretation: A contemporary introduction. Routledge.

[joap13096-bib-0032] Rosa, H. (2020). The uncontrollability of the world (J. C. Wagner, Trans.). John Wiley & Sons.

[joap13096-bib-0033] Sperry, L. (2025). Spiritually integrated psychotherapy: Infusing spirituality in clinical practice (3rd ed.). Taylor & Francis.

[joap13096-bib-0034] Stein, M. (2008). “Divinity expresses the self …” An investigation. Journal of Analytical Psychology, 53(3), 305–327. 10.1111/j.1468-5922.2008.00729.x 18494670

[joap13096-bib-0035] Stein, M. (2024). The faithless analyst. Journal of Analytical Psychology, 69(5), 874–883. 10.1111/1468-5922.13042 39326404

[joap13096-bib-0036] Trombley, C. (2024). Spirituality, religion, and recovery: What about the nonreligious? Journal of Substance Use, 1‐4. 10.1080/14659891.2024.2356571 39055109

[joap13096-bib-0037] Vogel, R. T. (2017). Individuation und Wandlung. Der “Werdensprozess der Seele” in der Analytischen Psychologie C. G. Jungs. Kohlhammer.

[joap13096-bib-0038] Wintering, N. A. , Yaden, D. B. , Conklin, C. , Alizadeh, M. , Mohamed, F. B. , Zhong, L. , Bowens, B. , Monti, D. A. , & Newberg, A. B. (2021). Effect of a one‐week spiritual retreat on brain functional connectivity: A preliminary study. Religions, 12(1), 23.

